# Validity and Reliability According to the Type of Examiners in the Process of Calibrating Dental Caries Experience Using the DMFT Index

**DOI:** 10.3390/mps7050083

**Published:** 2024-10-16

**Authors:** Anna Paola Fernández-Coll, María Claudia Garcés-Elías, Jorge A. Beltrán, Roberto A. León-Manco, Janett Mas-López

**Affiliations:** Facultad de Estomatología, Universidad Peruana Cayetano Heredia, Lima 150135, Peru; maria.garces@upch.pe (M.C.G.-E.); jorge.beltran@upch.pe (J.A.B.); roberto.leon@upch.pe (R.A.L.-M.); janett.mas@upch.pe (J.M.-L.)

**Keywords:** epidemiology, validity, reliability

## Abstract

The process of examiner calibration is an essential step in all epidemiological research, as it aims to ensure uniform interpretation, understanding, and application of the instrument to be used. This ensures that the data collected will be valid and reliable. This study aimed to determine the differences in concordance in dental caries calibration across three dental specialties. The population consisted of 45 dentists, divided into three groups: 15 general dentists working in the public sector, 15 dentists specializing in Dental Public Health, and 15 dentists specializing in Restorative and Aesthetic Dentistry. The calibration process was carried out in three stages: theory, calibration using photographs, and calibration on natural teeth, performed by the gold standard. In the first validity process, a statistical difference was only found between the Kappa values of the inter-examiner calibration process using photographs. For the evaluation of teeth, in the second validity process, 33.33% (n = 15) of the participants achieved “almost perfect agreement.” Finally, only 75.56% (n = 34) of the examiners were considered for the reliability report; of this group, 52.94% (n = 18) were in “almost perfect agreement,” and 35.29% (n = 12) were in “substantial agreement.” The validity and reliability of the dental caries experience calibration process did not present significant statistical differences between general dentists in the public sector, dentists specializing in Dental Public Health, and dentists specializing in Restorative and Aesthetic Dentistry.

## 1. Introduction

Despite being a chronic preventable disease, dental caries continues to pose significant challenges to global public health, maintaining high prevalence rates for over 25 years [1]. Designing effective preventive strategies tailored to the population’s needs necessitates evaluating the disease’s prevalence based on systematically, methodologically, and standardized data collection [2]. The most widely used method globally to determine the epidemiological profile of caries is the DMFT (decayed, missing, and filled teeth) of the World Health Organization (WHO), which records the disease as present when an unmistakable cavity is evident [3,4]. When data collection is conducted on a large scale, the involvement of multiple examiners becomes essential. Therefore, implementing a rigorous training and calibration process is crucial. This process ensures that the individuals responsible for data collection are aligned with the gold standard and with each other regarding the diagnostic criteria before the main study. By doing so, variability among examiners can be minimized, thereby guaranteeing the research’s quality and the reliability of the results [5,6]. Additionally, the reproducibility of the instrument is determined by its ability to achieve concordance with the results, which is reliably evaluated using the Kappa statistical method. The level of coherence in inter- and intra-examiner evaluations should ideally range between 85% and 95% [7]. It is important to highlight that the epidemiological information collected from such studies is fundamental for decision-making in both clinical and public health contexts. This information not only directly supports evidence-based treatment but also indirectly contributes to the formulation of guidelines and policies for health services at the national level [8]. In Peru, the availability of population-specific data is limited, outdated, and in high demand. Therefore, obtaining valid epidemiological data is crucial for designing policy strategies aimed at effectively improving the population’s oral health [9]. It is essential to establish new calibration methodologies to ensure the reliability of data used in decision-making. Additionally, calibration process studies primarily focus on experienced examiners, leaving a significant gap in our understanding of those with limited experience or different specialties [5]. Therefore, this investigation aimed to determine the differences in concordance in dental caries calibration across three dental specialties.

## 2. Materials and Methods

This descriptive study involved a population of 45 voluntarily participating dentists, divided into three groups: 15 general dentists working in the public sector, 15 dentists specializing in Dental Public Health, and 15 dentists specializing in Restorative and Aesthetic Dentistry. The purpose was to compare the results obtained among each group of interest. The volunteers were referred by the postgraduate unit of the Faculty of Stomatology at Universidad Peruana Cayetano Heredia, Lima, Peru (UPCH). The selection of these study groups was intentional: general dentists in the public sector are responsible for the national diagnosis of dental caries; specialists in Restorative and Aesthetic Dentistry are expected to possess the highest diagnostic capacity; and Dental Public Health specialists are assumed to have the least diagnostic capacity due to their lack of direct healthcare activities. Therefore, if similar results were found across the three groups, the applied methodology would be validated for use among different professional groups.

The postgraduate unit of the UPCH provided a list of students specializing in Dental Public Health and Restorative and Aesthetic Dentistry, as well as general dentists from the public sector who had received continuing education courses. Using this information, potential volunteers were selected non-probabilistically and invited via email to participate in the study voluntarily. The inclusion criteria were being a professional, and those who did not complete the calibration process were excluded. None of the selected participants had previously participated in a calibration process. The population distribution included 8 women and 7 men in each group, with an average age of 35.26 years (SD = 9.78) and professional experience ranging from 3 to 15 years. The specialty students were in their first and second years of postgraduate studies. For the dental caries experience calibration process, the DMFT Index was chosen, following the WHO criteria [7]: 1: tooth with caries, 2: tooth with restoration and caries, 3: tooth with restoration without caries, 4: tooth extracted due to caries, 5: tooth extracted for other reasons, 6: tooth with pit and fissure sealant, 7: bridge abutment, 8: not erupted, and 9: tooth not registrable.

## 3. Ethical Consideration

The Ethics Committee of the Universidad Peruana Cayetano Heredia approved the study on 12 September 2022, and all participants provided informed consent.

## 4. Procedure

For epidemiological studies on dental caries, the calibration process standard is outlined by the WHO in the document “Oral Health Surveys: Basic Methods”, which establishes two calibration stages: theoretical and practical, involving photographs and patients [7]. Based on this methodology, the authors propose the process described in the present article. The calibration process was developed in three stages: theoretical training, calibration using photographs, and calibration with natural teeth. These stages were conducted by the gold standard (GS) of the study, a specialist in Restorative and Aesthetic Dentistry with over 20 years of professional experience in the field. The theoretical stage involved a 30 min master class delivered virtually via Zoom^®^, where the DMFT Index and its criteria were explained, and participants’ questions were answered. The second stage, calibration using photographs, was performed virtually following the theoretical training. The GS selected 30 high-resolution photographs (minimum 1280 × 720 p) of teeth from the Restorative and Aesthetic Dentistry Service of the UPCH that met all the DMFT Index criteria. These photographs were coded and evaluated according to the DMFT Index criteria by the GS. The photographs were randomly divided into two groups of 15 images each and presented in a Microsoft PowerPoint 2021^®^ presentation, programmed to change automatically every 30 s. The first round of inter-examiner calibration was initiated to evaluate validity, with each examiner recording their answers in Google Forms^®^ (https://docs.google.com/forms/, accessed on 12 April 2024). At the end of the first round, the GS reviewed each case to address any remaining questions. This procedure was repeated for the second round of photographs, with all examiners evaluating the same images.

The final stage was conducted on natural permanent teeth donated by the Maxillofacial Surgery Service of the UPCH. For this, models of natural teeth were created separately. The gold standard (GS) selected teeth that met the diagnostic criteria of the DMFT Index, which were placed on plaster bases for proper handling, coding, and evaluation. The examiners performed two evaluation processes, each involving a group of 30 teeth with 30 s allocated per tooth. All examiners evaluated the same teeth and recorded their responses in Google Forms^®^. At this stage, no reinforcement from the GS was provided, focusing solely on inter-examiner calibration. The second process was critical for determining the validity of each examiner. Additionally, after an interval following the last process, the examiners re-evaluated the last randomly ordered group of teeth using the same procedure to conduct intra-examiner calibration and assess the reliability of the examiners. It is important to note that for this report, only the results of examiners who exceeded a Kappa coefficient value of 0.60 in their final process were considered. This conventional practice in calibration processes ensures that only examiners who demonstrated the minimum agreement with the GS in the inter-examiner calibration of validity are included (Appendix A).

## 5. Analyses and Statistics

The results from all calibration processes involving photographs and teeth, recorded in Google Forms^®^, were compiled into a single database using Microsoft Excel 2021^®^. This database included records from both the gold standard (GS) and the examiners. Subsequently, validity and reliability values were calculated using the Kappa coefficient (Kappa). These values were categorized according to WHO recommendations [6] into the following levels: “poor agreement”, “fair agreement” (0.21–0.40), “moderate agreement” (0.41–0.60), “substantial agreement” (0.61–0.80), and “almost perfect agreement” (0.81–1.00). The agreement levels were described in terms of absolute and relative frequencies. For the bivariate analysis, normality was assessed using the Shapiro–Wilk test. The Kruskal–Wallis test with the post hoc Mann–Whitney U test was applied based on the data distribution characteristics. ANOVA and Chi-square tests for linear trends were also conducted. The analysis was performed using the statistical software STATA v. 17.0, with a 95% confidence level and a *p*-value < 0.05.

## 6. Results

Among the 45 study subjects, the inter-examiner calibration process for photographs revealed that, in the first validity process, Kappa values ranged from a minimum of 0.04 to a maximum of 0.82. In the second process, Kappa values ranged from a minimum of 0.02 to a maximum of 1.00. In the inter-examiner calibration process for teeth, the validity of the first process had Kappa values ranging from a minimum of 0.20 to a maximum of 0.86, while in the second process, Kappa values ranged from a minimum of 0.21 to a maximum of 0.88. For the intra-examiner calibration process, only 34 examiners who exceeded a Kappa value of 0.60 in their final validity process were considered. In this group, Kappa values for reliability ranged from a minimum of 0.39 to a maximum of 0.94. A statistical difference was found only between the Kappa values of the inter-examiner calibration process in photographs in the first validity process, between the general dentists and the Dental Public Health group (*p* < 0.05), and between the Dental Public Health group and the Restorative and Aesthetic Dentistry group (*p* < 0.05) (Table 1). 

Similarly, Kappa values were categorized for each process. In the first validity process for photographs, 26.67% (n = 12) were classified as having “poor agreement,” while 24.44% (n = 11) achieved “substantial agreement.” In the second process, 28.89% (n = 13) reported “moderate agreement,” and the same percentage was recorded for “substantial agreement.” For the evaluation of teeth, the validity of the first process showed 40.00% (n = 18) in both “moderate agreement” and “substantial agreement.” In the second process, 42.22% (n = 19) achieved “substantial agreement,” and 33.33% (n = 15) reached “almost perfect agreement.” Finally, only 75.56% (n = 34) of the examiners were included in the reliability report. Among this group, 52.94% (n = 18) were classified as having “almost perfect agreement,” and 35.29% (n = 12) had “substantial agreement”. No significant association was found between the study variables (*p* > 0.05) (Table 2).

## 7. Discussion

Since examiners may vary in their oral and dental evaluations during data collection, the calibration process is a crucial step in epidemiological research. It aims to ensure uniform interpretation, understanding, and application of the instruments used, thereby ensuring that the collected data are valid and reliable. To assess validity, it is necessary to have an expert or gold standard evaluate at least 25 individuals, who will be examined by the team members. This process is known as inter-examiner calibration [7]. In contrast, reliability is assessed through intra-examiner calibration, which evaluates the consistency of each evaluator individually [8].

In the present study, no significant differences were found between the three groups analyzed, reinforcing the notion that any calibrated dentist, regardless of specialization, can reliably and validly collect epidemiological data. This suggests that the presence of specialists in dental caries is not essential for conducting the calibration process effectively. Similarly, Nabarrette et al. (2023) found in their epidemiological study that inexperienced examiners faced no difficulty in assessing the presence or absence of caries. These results may be attributed to the proximity of the theoretical and practical calibration stages; however, it should be noted that Kappa values decreased after three months, highlighting a challenge in maintaining reproducibility over time [10]. Conversely, Assaf et al. (2006) reported stable Kappa values over a 12-month period among experienced evaluators [11].

Regardless of the level of instruction, the calibration process is crucial and must adhere to the guidelines developed by the WHO throughout the entire phase [4]. However, despite these guidelines, the clinical phase, which involves examining individuals, has become a significant limitation due to time constraints and the scarcity of volunteer participants. Consequently, it is proposed to replace live examinations with mock-ups created from donated extracted natural teeth that meet the necessary criteria for applying the DMFT Index. Although extracted teeth do not possess the same characteristics as those in the oral cavity, the DMFT criteria are quite clear, as a cavity must be present to be diagnosed as caries. This criterion is not affected by the humidity of the dental specimens, making the use of these models feasible despite their limitations [7]. However, advancements in technology now allow for impressions that more closely replicate dental structures, representing the next step in improving the application of these technologies. 

Similarly, various tools used in epidemiological studies of oral health, such as the Community Periodontal Index (CPI) [12], the Dean Index for fluorosis [13], and the International Caries Diagnosis and Screening System (ICDAS) [14], could be adapted to the current proposal and undergo a calibration process using specimens and mock-ups. In relation to the ICDAS, Christian et al. (2017) found no significant difference between calibration processes conducted in vivo and those performed using photographs [15].

One limitation of this new approach to the calibration process is the need for improved standardization procedures. It is essential to use our own photographs for each study to ensure consistency. Additionally, the ability to maintain results over the long term was not evaluated, as the study was conducted at a single point in time without comparing results over an extended period. It is important to note that this calibration process is specific to the research group in question. It is recommended to continue this line of research and develop a new calibration process to ensure the consistency and validity of the obtained values.

Finally, in developing calibration protocols and strategies for epidemiological studies, the aim is to simplify the process for both experienced and inexperienced dentists while ensuring the collection of reliable data that accurately reflect the clinical status of the population. Although live physical assessments improve examiners’ ability to evaluate teeth and adapt to epidemiological conditions, practical challenges such as time constraints, resource requirements, and limited participant cooperation can arise. Therefore, there is a need to reduce volunteer requirements by utilizing specimens for examinations. As demonstrated in this study, the proposed approach is feasible, as it achieved acceptable levels of inter- and intra-examiner reliability.

## 8. Conclusions

The validity and reliability of the dental caries calibration process showed no significant statistical differences between general dentists working in the public sector, dentists specializing in Dental Public Health, and those specializing in Restorative and Aesthetic Dentistry.

## Figures and Tables

**Table 1:** Validity and reliability according to the type of examiners in the process of calibrating dental caries experience using the DMFT Index.

Examiners	N	Photographs								Teeth											
		Inter-Examiner								Inter-Examiner								Intra-Examiner			
		Validity 1				Validity 2				Validity 1				Validity 2				Reliability			
		Mean	SD	Min	Max	Mean	SD	Min	Max	Mean	SD	Min	Max	Mean	SD	Min	Max	Mean	SD	Min	Max
GD	15	0.54 a	0.24	0.05	0.82	0.59 a	0.26	0.03	1.00	0.52	0.18	0.20	0.73	0.63	0.19	0.21	0.88	0.83	0.12	0.64	0.94
DPH	15	0.13 ab	0.07	0.04	0.22	0.24 ab	0.25	0.02	1.00	0.56	0.17	0.20	0.81	0.74	0.19	0.21	0.88	0.78	0.13	0.39	0.94
RAD	15	0.57 b	0.11	0.38	0.74	0.62 b	0.09	0.48	0.81	0.60	0.13	0.34	0.86	0.69	0.10	0.50	0.83	0.76	0.16	0.51	0.94
Total	45	0.41	0.26	0.04	0.82	0.48	0.27	0.02	1.00	0.56	0.16	0.20	0.86	0.69	0.17	0.21	0.88	0.79	0.14	0.39	0.94
*p*		<0.001 *				<0.001 *				0.437 *				0.211 *				0.369 **			

GD: General dentist. DPH: Dental Public Health. RAD: Restorative and Aesthetic Dentistry. N: number of examiners. SD: standard deviation. Min: minimum. Max: maximum. *p*: statistical significance. * Kruskal–Wallis test with post hoc Mann–Whitney U test, identical lowercase letters indicate a statistically significant difference (*p* < 0.001). ** ANOVA test.

**Table 2:** Categorization of validity and reliability according to the type of examiner in calibrating dental caries experience using the DMFT index.

Examiners		N	Photographs				Teeth					
			Inter-Examiner				Inter-Examiner				Intra-Examiner	
			Validity 1		Validity 2		Validity 1		Validity 2		Reliability	
			n	%	n	%	n	%	n	%	n	%
General dentist												
	Poor agreement (<0.20)	15	2	13.33	1	6.67	2	13.33	0	0.00	0	0.00
	Fair agreement (0.21–0.40)		1	6.67	1	6.67	2	13.33	2	13.33	0	0.00
	Moderate agreement (0.41–0.60)		5	33.33	6	40.00	4	26.67	4	26.67	0	0.00
	Substantial agreement (0.61–0.80)		4	26.67	3	20.00	7	46.67	6	40.00	2	22.22
	Almost perfect agreement (0.81–1.00)		3	20.00	4	26.67	0	0.00	3	20.00	7	77.78
Dental Public Health												
	Poor agreement (<0.20)	15	10	66.67	9	60.00	1	6.67	0	0.00	0	0.00
	Fair agreement (0.21–0.40)		5	33.33	4	26.67	1	6.67	2	13.33	1	7.69
	Moderate agreement (0.41–0.60)		0	0.00	1	6.67	7	46.67	0	0.00	0	0.00
	Substantial agreement (0.61–0.80)		0	0.00	0	0.00	5	33.33	3	20.00	6	46.15
	Almost perfect agreement (0.81–1.00)		0	0.00	1	6.67	1	6.67	10	66.67	6	46.15
Restorative and Aesthetic Dentistry.												
	Poor agreement (<0.20)	15	0	0.00	0	0.00	0	0.00	0	0.00	0	0.00
	Fair agreement (0.21–0.40)		3	20.00	0	0.00	1	6.67	0	0.00	0	0.00
	Moderate agreement (0.41–0.60)		5	33.33	6	40.00	7	46.67	3	20.00	3	25.00
	Substantial agreement (0.61–0.80)		7	46.67	8	53.33	6	40.00	10	66.67	4	33.33
	Almost perfect agreement (0.81–1.00)		0	0.00	1	6.67	1	6.67	2	13.33	5	41.67
Total												
	Poor agreement (<0.20)	45	12	26.67	10	22.22	3	6.67	0	0.00	0	0.00
	Fair agreement (0.21–0.40)		9	20.00	5	11.11	4	8.89	4	8.89	1	2.94
	Moderate agreement (0.41–0.60)		10	22.22	13	28.89	18	40.00	7	15.56	3	8.82
	Substantial agreement (0.61–0.80)		11	24.44	11	24.44	18	40.00	19	42.22	12	35.29
	Almost perfect agreement (0.81–1.00)		3	6.67	6	13.33	2	4.44	15	33.33	18	52.94
*p*			0.888 *		0.786 *		0.243 *		0.432 *		0.083 *	

N: Number of examiners. n: absolute frequency. %: relative frequency. *p*: statistical significance. * Chi-square test for linear trend.

## Data Availability

Data are available upon reasonable request from the corresponding author.

## Appendix A

Procedure flowchart
